# Noncontact optical device for measuring blood glucose in aqueous humor: a pilot clinical study investigating correlation with blood glucose levels

**DOI:** 10.1117/1.JBO.29.4.047001

**Published:** 2024-04-18

**Authors:** Eugene Yu-Chuan Kang, Chia-Rui Shen, Xin-Cheng Huang, Chun-Ya Kang, Tzu-Yi Lin, Wei-Hsin Hong, Lan-Yan Yang, Wei-Chi Wu, Yih-Shiou Hwang

**Affiliations:** a Chang Gung Memorial Hospital, Linkou Medical Center, Department of Ophthalmology, Taoyuan, Taiwan; b Chang Gung University, College of Medicine, School of Medicine, Taoyuan, Taiwan; c Chang Gung University, Graduate Institute of Clinical Medical Sciences, Taoyuan, Taiwan; d Columbia University Irving Medical Center, Vagelos College of Physicians and Surgeons, Department of Ophthalmology, New York, United States; e Chang Gung University, College of Medicine, Department of Medical Biotechnology and Laboratory Science, Taoyuan, Taiwan; f Taipei Medical University Hospital, Department of Education, Taipei, Taiwan; g Chang Gung Memorial Hospital, Clinical Trial Center, Taoyuan, Taiwan; h Jen-Ai Hospital Dali Branch, Department of Ophthalmology, Taichung, Taiwan; i Xiamen Chang Gung Memorial Hospital, Department of Ophthalmology, Xiamen, China

**Keywords:** glucose, monitoring, noncontact, optical, aqueous humor, blood, diabetes

## Abstract

**Significance:**

Monitoring blood glucose levels is crucial for individuals with diabetes. Noninvasive methods for measuring serum glucose levels have been explored to aid in blood glucose control for diabetes management.

**Aim:**

We introduced a noncontact optical glucometer (NCGM) for measuring glucose levels in the aqueous humor of the human eye. We also investigated the correlation between glucose levels in the NCGM and the aqueous humor, blood samples, and self-monitoring blood glucose devices.

**Approach:**

The optical system used in this study measured both the near-infrared absorption and polarized rotatory distribution of glucose molecules in the human aqueous humor. This prospective study’s outcomes were eye aqueous glucose level, preoperative blood glucose level, intraoperative blood glucose level, and NCGM reading of patients in a single center in Taiwan.

**Results:**

The NCGM’s measurements showed a strong correlation with blood glucose levels (intra-class correlation [ICC]: 0.95 to 0.98) and aqueous humor glucose levels (ICC: 0.76), indicating its ability to noninvasively measure blood glucose levels in human subjects.

**Conclusions:**

This NCGM may offer a convenient, pain-free, and rapid tool for measuring blood glucose levels in diabetic patients. The device could represent a significant advancement in noncontact hybrid optical glucose measurement systems.

## 1 Introduction

Diabetes mellitus (DM) is a prevalent and chronic disease that affected ∼586 million individuals worldwide in 2021, and this number is predicted to increase to 783 million by 2045.^1^ The increase in DM can be attributed to a lack of exercise, increased high-calorie food intake, obesity, and an aging population.^2^^,^^3^ Furthermore, the presence of DM can cause various systemic diseases.^4^^,^^5^ As such, individuals with DM are at greater risk of mortality, particularly from vascular complications.^6^^–^^8^

DM results in high blood glucose levels, increasing the risks of heart disease, high blood pressure, nephropathy, neuropathy, retinopathy, and stroke.^3^ The Collaborators on Trials of Lowering Glucose (CONTROL) group showed that intensive management of glucose levels in patients with DM significantly reduced major microvascular and macrovascular events.^9^^–^^11^ However, intensive management also significantly increased the incidence of hypoglycemia, which poses an immediate threat to mortality and morbidities, such as confusion and loss of consciousness, cognitive impairment, and an augmented propensity for cardiovascular events.^10^^,^^12^ Therefore, careful blood glucose monitoring is critical in managing patients with DM.^13^ Although measurement frequency depends on the clinical situation, patients with DM often require continual blood glucose monitoring. Therefore, the recommendation is for the self-monitoring of blood glucose (SMBG) in patients, which is now widespread.^13^

Current SMBG devices require a blood sample obtained by a finger prick and a test strip. The device then determines the blood glucose level and reports a reading. However, such devices can affect SMBG compliance due to the inconvenience, financial cost of the test strips, and pain and complications that can arise during sampling.^14^ Continuous glucose monitoring (CGM) devices have been developed to improve compliance and overall disease management.^15^^–^^18^ Although CGM overcomes the disadvantages of SMBG, patient compliance when using CGM is much lower.^14^

In recent years, there has been a growing interest in noninvasive (NI) methods for measuring glucose levels that employ a range of techniques including electrochemical and optical approaches.^19^^,^^20^ These advancements have led to the development of various device designs, such as sensors for tears and saliva, transdermal glucometers, and optical biosensors.^21^^–^^23^ Notably, the use of near-infrared (NIR) light in some of these NI glucometer designs has shown great promise due to its capability for contactless and precise detection.^19^ Therefore, this study aims to develop a novel, comfortable, convenient NI glucometer and validate it by testing blood glucose levels in diabetic and control human participants. The noncontact glucometer (NCGM) uses concurrent measurements of the NIR absorption and polarized rotatory distribution of glucose molecules in the eye’s aqueous humor to determine blood glucose levels. The accuracy of this device has been previously demonstrated in animal experiments and published in the literature.^24^ In this study, the NCGM’s measurements were validated in human subjects by correlating them with aqueous humor and blood glucose levels and SMBG readings.

## 2 Material and Methods

### 2.1 NCGM Device

**Fig. 1:**
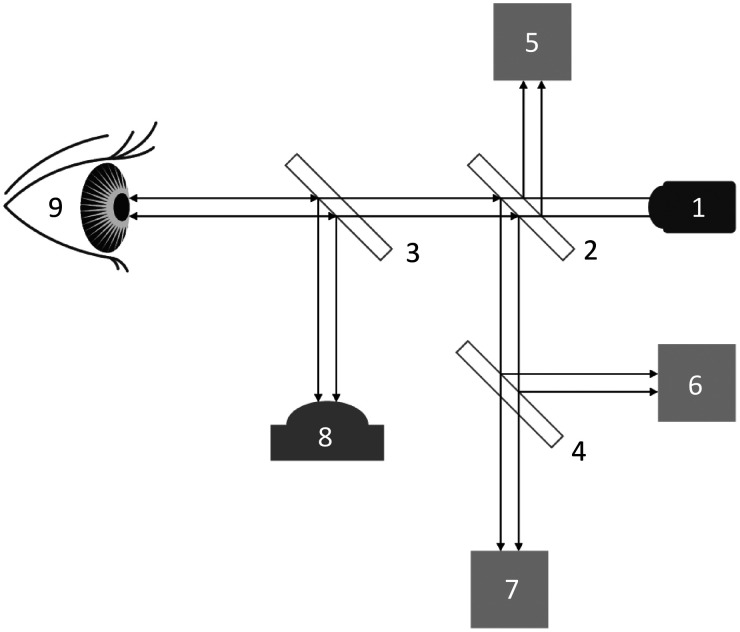
Diagram of the NCGM system including (1) laser diode, (2) to (4) beam splitters, (5) detector for optical feedback control, (6) detector for polarization of light, (7) detector for absorption of light, (8) monitoring camera, and (9) testing human eye.

The NCGM system, developed by Taiwan Biophotonic Corporation in Hsinchu, Taiwan, incorporates an advanced optical measurement setup comprising a light source, beam splitters, photodetectors, and a processing camera unit, as shown in Fig. 1. Utilizing a 1650 nm Fabry–Perot laser diode for its light source, the system adheres to the International Electrotechnical Commission’s safety standards for medical laser devices. The initial beam splitter focuses the laser light into the eyeball. The light that reflects off the eye’s anterior chamber is then redirected through the beam splitters to the photodiode detectors. The system is also equipped with a zeroth light receiving module, responsible for assessing the feedback light beam’s power intensity and estimating the emitted collimated light beam’s strength from the laser source, ensuring both safety and the prevention of analyte damage.

The light beam, originally emitted, is converted into a collimated beam by a collimator, proceeding through the first beam splitter to the analyte. A part of this beam is redirected as a feedback light beam to the zeroth receiving module, where it is converted into electrical signals. Post interaction with the analyte, the measurement light beam is split by the second beam splitter into two detection beams. These beams are then captured by the first and second light receiving modules. The crucial integration and collaboration between the optical measurement module, microprocessor, and light source underpin the functionality of this sophisticated glucose monitoring system.

These modules gather data on absorption energy and polarized optical rotatory distribution, aiding in the differentiation between emitted and reflected light. The device’s developer has identified optical angular differences and absorption energy differences across various biological molecules, including glucose, cholesterol, and uric acid. The processing unit leverages this information, employing simultaneous equations based on absorption and polarization values to calculate the biological molecules’ concentrations. Polynomial equations have been formulated to accurately depict the relationship between these concentrations and the measured optical and energy differences, taking into account both the target and interfering molecules. This analytical method enables the accurate determination of target molecule concentrations, even in the presence of interfering substances, by utilizing optical angular difference and absorption energy difference values. Ultimately, this system is capable of calculating glucose levels in the aqueous humor, showcasing its precision in monitoring biological molecules.

The general time frame to obtain a measurement is between 30 and 60 s. Further details about the device and glucose calculation can be found in the previously published literature^24^ and the US patents (Nos. US20160299058A1 and US9662004B2). The blood glucose level, which correlates with the aqueous humor glucose level, can then be determined. The device must be calibrated for each patient because each eye has a unique background optical signal. A blood glucose sample is taken simultaneously with the NCGM measurement to calibrate the device.

### 2.2 *In Vitro* Repeatability Tests of the NCGM

*In vitro* and repeatability experiments utilized custom-designed glass test tubes featuring convex bottoms that closely resembled the cornea’s curvature radius (r=6.4  mm) and a thickness of 500  μm. Seven glucose concentrations (50, 100, 200, 300, 400, 500, and 600  mg/dL) were prepared in a balanced salt solution (Alcon Laboratories Inc., Fort Worth, Texas) and added to the test tubes to simulate the eye’s anterior segment. The NCGM was then calibrated using distilled water. Following this, the absorption and polarization of the seven glucose solutions were measured with the NCGM. These measurements were recorded and graphed. Adhering to the ISO 15197:2013 standard, which standardizes *in vitro* glucose measurement, the measurements need to be within 15% of the target glucose value (when the target glucose value≥100  mg/dL) or 15  mg/dL (when the target glucose value<100  mg/dL).

### 2.3 Participants

This prospective study was conducted at a single center according to the Declaration of Helsinki and with the approval of the Institutional Review Boards at Taiwan Food and Drug Administration and Chang Gung Memorial Hospital (IRB No.: 201301153A0). This study was also registered with ClinicalTrials.gov (Identifier No.: NCT02430441). Patients planning to receive intraocular surgery, such as cataract surgery, intraocular injection, or vitrectomy, were invited to participate in the study. The study participants provided written patient informed consent after a detailed explanation, and informed consent was obtained from all subjects involved in the study. Participants were aged ≥20 years at the time of the study, with DM or not. Eligibility inclusion and exclusion criteria are provided in Table 1.

**Table 1:** Participant eligibility inclusion and exclusion criteria.

Inclusion	1. Age ≥20 years
	2. Able to provide a signed consent form
	3. Planned for ocular surgery (e.g., intravitreal injection, cataract surgery, or vitrectomy)
	4. At least one eye with a best-corrected visual acuity better than 20/200
Exclusion	1. An eye with corneal opacity
	2. History of any ocular surgery on the study eye within the previous 4 months or anticipated within 6 months after study enrollment
	3. History of retinal photocoagulation in the 4 months prior to study enrollment
	4. Evidence of ocular infection or uveitis
	5. Vitamin C use
	6. For women of child-bearing potential: pregnant or lactating or intending to become pregnant within 6 months
	7. A condition that, in the opinion of the investigator, would preclude participation: e.g., unstable medical status, such as high blood pressure; unstable or uncontrolled major organ diseases, such as cardiovascular, liver, blood, lung, and cerebrospinal diseases; and other morbidities that may interfere with the study results
	8. Intolerable to the blood collection procedure
	9. Alcohol or drug abuse

### 2.4 Study Protocol

For each enrolled participant, one eye was assessed (the planned surgery eye or one eye if receiving two-eye surgery). The study’s outcomes were eye aqueous glucose level, preoperative blood glucose level, intraoperative blood glucose level, and NCGM reading. Patients fasted for 8 h before the experiment. The experimental protocol is described in Table 2. All adverse events were reported and coded based on the Medical Dictionary for Regulatory Activities. Patient hospital medical records were examined.

**Table 2:** Experimental protocol.

Step		
Participants fasted for 8 h		
Device (NCGM) warm-up		
Collection of participant’s information		
Planned surgery preparation		
**Time (min)**	**Experimental process**	
−30	Collect blood sample	SMBG measurement
−10	NCGM measurement	SMBG measurement
	**Planned surgery began**	
0	Aqueous tapping from the NCGM test eye	
	Collect blood sample	SMBG measurement
	**Experiment end**	

NCGM, noncontact glucometer; SMBG, self-monitoring of blood glucose.

### 2.5 Aqueous Tapping

An aqueous sample was collected prior to the planned surgical procedures. The preoperative sampling of aqueous fluid involved topical or periocular anesthesia and 5% povidone-iodine using a sterile lid speculum. A 30-gauge 1-in. needle was used for anterior chamber paracentesis. Approximately 0.1 mL of aqueous humor was collected during sampling. Samples were transferred to microcentrifuge tubes and were assayed for glucose levels immediately.

### 2.6 Glucose Measurement Methods

The glucose levels in aqueous samples from the tapping solution were determined using the hexokinase/glucose-6-phosphate dehydrogenase method with deproteinization, which is widely acknowledged as the standard reference method for measuring glucose (Fisher Diagnostics, Middletown, Virginia).^25^ Serum glucose levels were assessed using two different assays: one based on venous blood samples and the other through SMBG measurements. For the venous blood samples, 2 mL of blood was drawn through phlebotomy and immediately sent for analysis. The serum glucose concentration in these samples was also measured using the hexokinase/glucose-6-phosphate dehydrogenase method with deproteinization, adhering to the aforementioned reference method for glucose measurement (Fisher Diagnostics, Middletown, Virginia). For the SMBG measurement, finger strips were used with SMBG devices according to the manufacturer’s instructions (Roche Diagnostics Corporation, Indianapolis, Indiana).

### 2.7 Statistical Analysis

In this study, two approaches were employed to evaluate the agreement between NCGM measurements and aqueous glucose, blood glucose, and SMBG measurements. The first approach involved calculating the intraclass correlation (ICC) of consistency between the two methods. The ICC value was determined using a two-way mixed-methods model, with observations as a random effect and the raters (the two measurement methods) as a fixed effect. An ICC value of >0.7 signified good agreement between the two measurement methods. To assess the acceptability of individual differences between the methods, the acceptable boundaries were established at the 95% confidence interval (CI) of the mean difference between them. A two-sided P-value of <0.05 was considered statistically significant. The second approach involved generating a Bland–Altman plot of the mean glucose levels from NCGM in comparison with other measurements against their difference. The Bland–Altman plot and ICC analysis were conducted using MedCalc Statistical Software (version 13.1.2.0; MedCalc Software, Ostend, Belgium). These methods were essential in ensuring the precision and dependability of the measurements.

## 3 Results

### 3.1 Study Participants

This study enrolled 28 participants between April of 2015, and December of 2017. The participants’ mean age was 66.38 years; 15 (53.6%) were female, and all were Taiwanese inhabitants. Eight (28.6%) participants had known DM (all were type 2). Patients’ fasting blood glucose levels were as follows: 9 (32.1%) <100  mg/dL, 11 (39.3%) 100 to 150  mg/dL, and 8 (28.6%) >150  mg/dL (maximum 320  mg/dL). All participants completed a 6-month clinical follow-up. There were no subjective or objective findings related to optical damage to the external eye, lens, or fundus due to the measurements or systemic adverse events.

### 3.2 *In Vitro* Repeatability Test

**Fig. 2:**
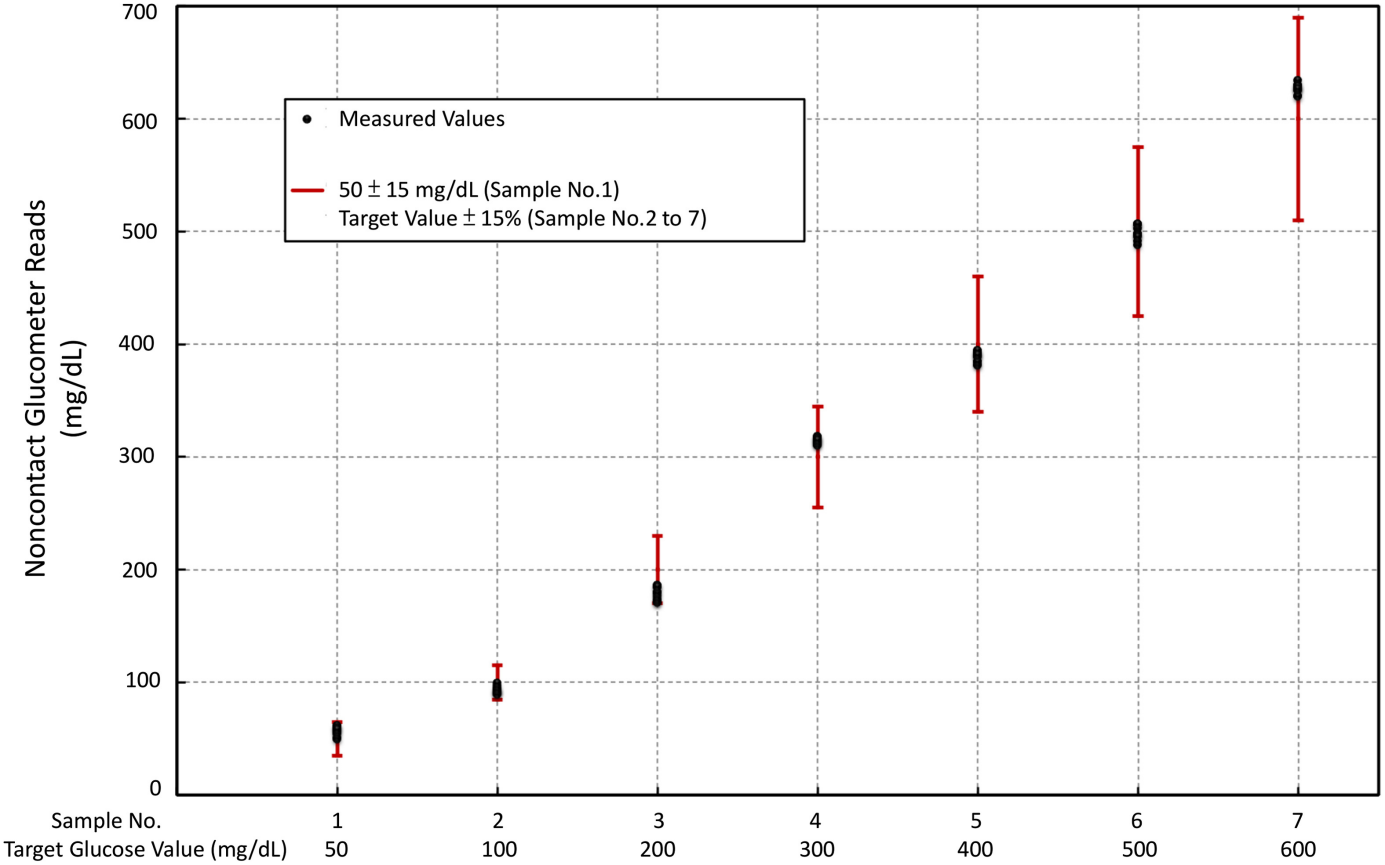
Repeatability test of the NCGM system using seven glucose concentrations (50, 100, 200, 300, 400, 500, and 600  mg/dL).

Prior to applying the NCGM to human subjects, the device was tested with standardized glucose concentrations to ensure its repeatability and accuracy *in vitro*. Employing the methods outlined in the previous section, each glucose solution underwent 12,000 measurements (10 times per second for 20 min). Subsequently, consecutive sets of 1000 measurements were averaged, and the results are shown in Fig. 2. The red lines in Fig. 2 indicate the error limits set by ISO standards: ±15  mg/dL for a glucose concentration of 50  mg/dL and an error margin limited to within ±15% for concentrations ranging from 100 to 600  mg/dL. The black dots represent the measurements obtained from our device, which consistently fell within these prescribed error margins across the range of standard glucose level solutions tested.

### 3.3 Correlation Between the Different Measurements

**Fig. 3:**
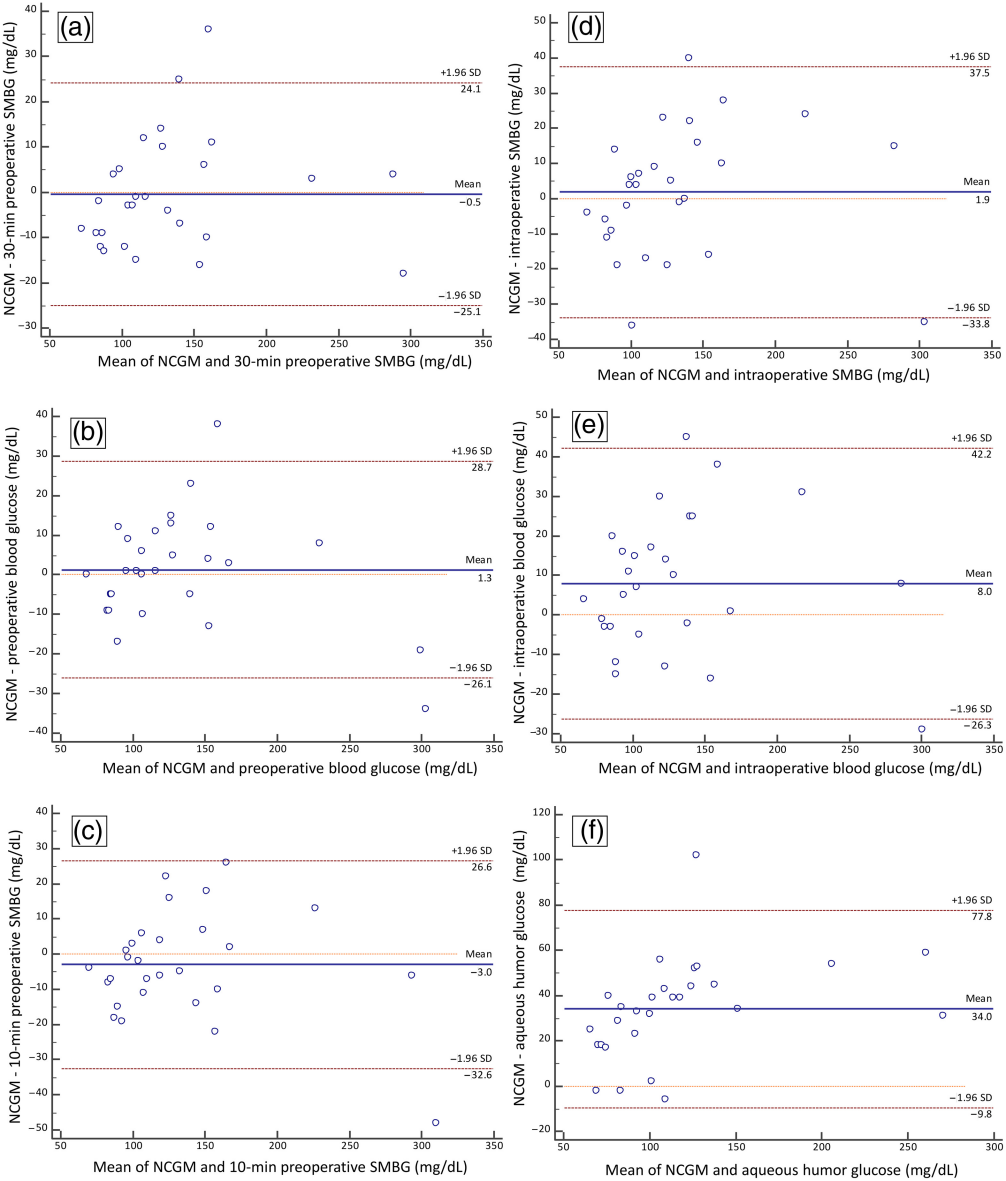
Bland–Altman plot demonstrating the agreement of NCGM with (a) 30-min preoperative SMBG, (b) preoperative blood glucose, (c) 10-min preoperative SMBG, (d) intraoperative SMBG, (e) intraoperative blood glucose, and (f) aqueous humor glucose.

**Fig. 4:**
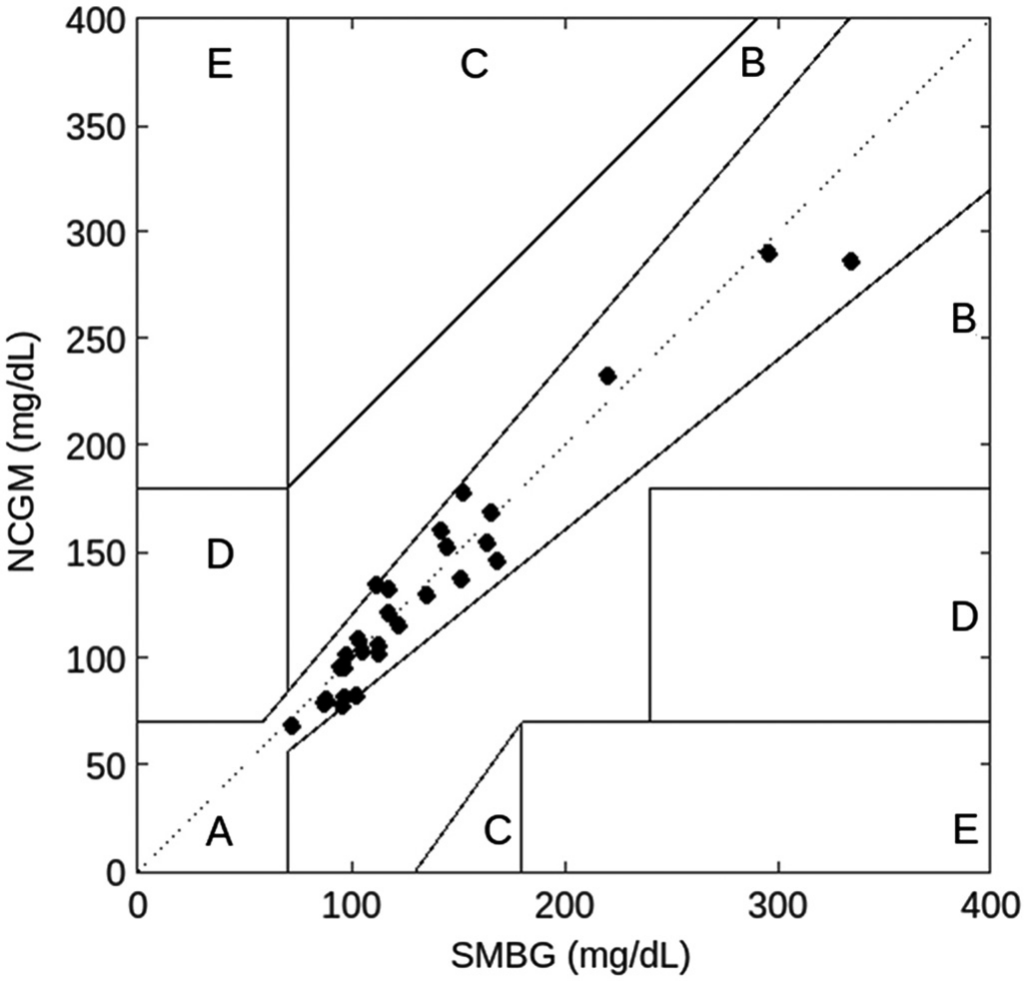
Clarke’s error grid analysis comparing measurements from the NCGM with SMBG values.

The ICC results are shown in Table 3. The ICCs of NCGM to SMBG or blood glucose levels were all greater than 0.9 preoperatively and intraoperatively, indicating a statistically significant correlation (all P<0.001). Furthermore, the ICCs of aqueous humor to SMBG or blood glucose levels were ∼0.8. In addition, the ICC of NCGM to aqueous humor glucose levels was 0.76. The agreement of NCGM to other measurements and aqueous humor glucose was also evident in the Bland–Altman plots (Fig. 3). Most of the observations in these plots did not exceed the upper and lower 95% CI boundaries, indicating that the individual differences between the measurements were within the acceptable range and did not deviate significantly from the mean difference between measurements. We also utilized Pearson’s linear correlation coefficient to assess the relationship between different measurements. Our analysis revealed a significant linear correlation between NCGM reading and blood glucose (R=0.968, p<0.001). Moreover, there was a notable correlation between aqueous glucose and blood glucose (R=0.954, p<0.001). These results indicate a strong correlation between NCGM readout from aqueous glucose and blood glucose levels, suggesting a reliable prediction of blood glucose levels by aqueous glucose levels. Furthermore, a Clarke error grid analysis was conducted and is shown in Fig. 4 to assess the accuracy of the NCGM system. SMBG values served as the reference for this analysis. All data points fall within zone A, demonstrating a minimal risk of error in the measurements.^26^ These findings affirm the validity and reliability of the measurements and instill confidence in the accuracy of the NCGM results.

**Table 3:** The results of intra-class correlation between different measurement methods.

Variable	ICC (95% CI)
NCGM versus	
30-min preoperative SMBG	0.976 (0.949 to 0.989)
Preoperative blood glucose	0.973 (0.942 to 0.987)
10-min preoperative SMBG	0.967 (0.930 to 0.984)
Intraoperative SMBG	0.949 (0.894 to 0.976)
Intraoperative blood glucose	0.947 (0.874 to 0.976)
Aqueous humor glucose versus	
30-min preoperative SMBG	0.780 (−0.059 to 0.944)
Preoperative blood glucose	0.800 (−0.046 to 0.947)
10-min preoperative SMBG	0.760 (−0.064 to 0.936)
Intraoperative SMBG	0.788 (−0.051 to 0.943)
Intraoperative blood glucose	0.841 (0.046 to 0.956)
NCGM versus aqueous humor glucose	0.758 (−0.046 to 0.930)

NCGM, noncontact glucometer; min, minute; SMBG, self-monitoring of blood glucose; ICC, intra-class correlation.

## 4 Discussion

This study aims to assess the NCGM, utilizing polarization and absorption signals to estimate glucose levels in the eye’s aqueous humor in the human subjects. From our study, NCGM demonstrated strong correlations with SMBG readings and glucose levels in both aqueous humor and blood. Optical glucose measurement in aqueous humor has been explored since the 1980s, it but lacked comprehensive human studies and correlation with blood glucose levels. The results of the NCGM represent a significant advancement in noncontact optical glucose monitoring, showing a promising efficacy for glucose monitoring in DM patients.

Aqueous humor is produced in the ciliary process, which is supplied by the major arterial circle that originates entirely from long posterior ciliary arteries.^27^ Aqueous humor production depends on active secretion, diffusion, and ultrafiltration.^28^ Numerous studies have demonstrated a strong association between aqueous humor and blood glucose levels.^29^^,^^30^ Despite a slight difference in glucose levels between blood and aqueous humor, the ratio remains constant, as observed in our study. Given this consistent ratio between aqueous humor and blood glucose, we can utilize the NCGM to determine serum glucose levels in humans. Our laboratory has previously demonstrated that NCGM readings show a strong correlation with aqueous humor and blood glucose levels in rabbits, with an average aqueous humor to blood glucose ratio of 0.82 before the glucose challenge.^24^ In the current study with human participants, the NCGM measurements demonstrated strong predictive accuracy for blood glucose levels (ICC>0.9 with P<0.001). The correlation between NCGM and SMGB at various time points was also high (ICC>0.9 with P<0.001). The NCGM device holds the potential to supplement conventional blood testing methods in monitoring glucose levels for individuals with diabetes.

Over the last two decades, several NI glucose monitoring technologies have been introduced.^23^ These methods include cutaneous and subcutaneous measurements using sweat and saliva.^31^ Although these techniques avoid using blood samples, they still require single-use strips. A contact lens with a built-in glucose-sensing electrical circuit has also been reported, but its accuracy could be affected by air humidity.^32^^,^^33^ Both biochemical and optical detection methods are commonly used in NI glucose monitoring techniques. Biochemical assays measure changes in biochemical reactions using reagents sensitive to glucose,^34^ and optical methods rely on glucose’s intrinsic molecular properties to determine its levels in tissue fluids.^35^^–^^40^ The main challenge that NI glucose monitoring techniques face is achieving the required accuracy at an affordable cost without using blood samples for measurements.^41^ The majority of NI glucose monitoring techniques utilized a single method to monitor physiological changes related to blood glucose levels. However, these measurements could be influenced by various other physiological factors, resulting in reduced sensitivity and specificity. Implementing a multi-sensing approach may help minimize errors associated with using only one method, ultimately enhancing the overall accuracy of glucose readings compared with relying on a single sensing technology.

Since Rabinovitch et al. introduced the NI measurement of glucose in the eye’s aqueous humor in the 1980s,^42^ using polarized light to detect glucose-induced rotation angles, this method has gained popularity. Aqueous humor, a clear fluid in the eye’s anterior chamber, enables such measurements due to the transparency of the cornea, although corneal birefringence complicates the process by causing light refraction and scattering. Despite being an ultrafiltrate of blood with similar electrolyte concentrations^43^ but significantly lower protein levels and higher ascorbate levels, aqueous humor’s glucose, lactate, and urea levels are about 80% of those in plasma.^44^ Because lactate, ascorbate, and urea are optically active molecules, they can interfere with glucose measurements using the polarization technique.^45^ Although various techniques aim to improve measurement stability and sensitivity, commercial availability of NI glucose monitoring devices remains limited due to issues such as corneal birefringence and the potential for time delays between blood and aqueous humor glucose levels. Our study’s NCGM technique, with a measurement timeframe of 30 to 60 s and the ability to gather multiple optical data points simultaneously, including optical angular and absorption energy differences, stands out for its use of polynomial equations to calculate concentrations, which offers a significant advancement in the field.

Our secondary findings indicate that the NCGM exhibits higher correlations with other measurements (ICC>0.9) compared with the correlation of aqueous humor glucose levels with other metrics (ICC = 0.8). This trend could be attributed to several factors. Primarily, the variability in glucose concentrations within the aqueous humor might surpass what is detected by other methods, likely due to biological fluctuations or sampling techniques, accounting for the lower ICC in aqueous humor measurements.^46^ Nonetheless, it is crucial to highlight the established correlations between glucose levels in the aqueous humor and blood glucose concentrations.^47^ The NCGM system, calibrated using blood glucose levels before measurements, achieves greater consistency in predicting blood glucose levels from the aqueous humor glucose concentrations.

Some other concerns and limitations exist regarding the device. First, a few measurements fell outside the 95% CI boundaries in our study, which could be due to the variability in operational conditions or simply by chance (values beyond the 95% CI). Thus, in future clinical applications, it may be essential to perform repeated measurements to ensure the highest accuracy, similar to the approach taken with other medical devices. Calibration was required for the optical measurement of aqueous glucose. In this study, the NCGM device used a single-person calibration approach, which required one simultaneous blood glucose measurement when first using the device. Aqueous humor glucose levels were not used as a calibration reference because they were not accessible to patients for device recalibration. In addition, previous studies have indicated a 5 to 30-min time delay in glucose diffusion to aqueous humor in rabbits after a glucose challenge.^47^^,^^48^ Thus, the participants in this study were asked to fast for 8 h before sampling to minimize potential bias. Furthermore, it is advised to wait between 5 and 30 min after a glucose challenge before using the NCGM for more accurate results. Aqueous humor is a transparent fluid containing various molecules, electrolytes, and proteins.^49^ Therefore, optical interferences from these molecules should be considered. Among the molecules present in the aqueous humor, only vitamin C was found to potentially interfere with polarization recording. Consequently, candidates regularly taking vitamin C supplements were excluded from this study. For polarization recording, tissues or organs with low scattering are needed. The eye is suitable for noncontact glucose measurements due to the lack of scattering.^50^ However, several factors can impact polarimetric ocular measurements, including corneal birefringence, rotation, and eye motion artifacts.^51^^,^^52^ To address these factors, NCGM measurements were collected within seconds to avoid eye motion artifacts. Finally, it is important to note that corneal pathology may also impact the measurements. This study excluded patients with known corneal diseases. The applicability of the NCGM in patients with corneal diseases may require further investigation.

## 5 Conclusion

This study demonstrated the capabilities of the noninvasive, noncontact hybrid optical glucose monitor in accurately measuring glucose levels in the aqueous humor of the human eye. The NCGM measurement exhibited a strong correlation between aqueous humor and blood glucose level. The NCGM device has the potential to complement traditional blood testing methods in managing glucose levels for diabetic patients.

## Data Availability

The data that support the findings of this article are not publicly available due to the data security policy of Chang Gung Memorial Hospital. They can be requested from the corresponding author.
